# Nonlinear Modeling and Differential-Voltage Control of an Electrostatic MEMS Micromirror for Miniaturized Laser Communication Terminals

**DOI:** 10.3390/mi17060753

**Published:** 2026-06-22

**Authors:** Xuan Wang, Chen Wang, Meilin Xie, Zengxin Liu, Junfeng Han

**Affiliations:** 1 Key Laboratory of Space Precision Measurement Technology, Chinese Academy of Sciences, Xi’an 710119, China; wangx002@outlook.com (X.W.);; 2 Xi’an Institute of Optics and Precision Mechanics, Chinese Academy of Sciences, Xi’an 710119, China; 3 College of Information and Control Engineering, Xi’an University of Architecture and Technology, Xi’an 710055, China; zxliu@xauat.edu.cn

**Keywords:** MEMS micromirror, electrostatic actuation, differential-voltage control, nonlinear modeling

## Abstract

Electrostatic MEMS micromirrors provide a compact and low-power beam-steering solution for miniaturized laser communication terminals. However, when they are used for quasi-static beam pointing rather than resonant scanning, the nonlinear voltage–angle relationship, bidirectional actuation asymmetry, and terminal-level installation errors can significantly degrade pointing accuracy. In this paper, a nonlinear modeling and differential-voltage control method is investigated for a two-axis electrostatic MEMS micromirror used in a miniaturized laser communication terminal. The device under test is a bonded aluminum MEMS micromirror with a 5.0 mm aperture. Static and dynamic characterization results show that the micromirror achieves maximum mechanical deflection angles of 5.215° and 5.161° along the X and Y axes, respectively, with resonant frequencies of 317 Hz and 319 Hz. To improve the accuracy of quasi-static pointing, the differential-voltage actuation principle is analyzed, and a nonlinear voltage–angle model is established based on measured deflection data. Compared with a first-order linear model, the cubic nonlinear model reduces the root-mean-square fitting error from 0.142° to 0.0127° for the X axis and from 0.132° to 0.0109° for the Y axis. Furthermore, a terminal-level calibration architecture based on a quadrant detector is introduced to map the MEMS angular deflection to the received spot position. The proposed modeling and calibration approach provides an actuator-level basis for accurate beam pointing and closed-loop acquisition in miniaturized laser communication terminals.

## 1. Introduction

Free-space optical communication has attracted increasing attention for satellite networks, airborne platforms, and mobile optical terminals because of its high data rate, narrow beam divergence, and strong anti-interception capability [1,2,3,4,5,6]. Compared with conventional radio-frequency links, optical communication can provide higher antenna gain and larger communication capacity with a smaller aperture. However, these advantages are achieved only when the optical beam is accurately pointed toward the receiving terminal. Therefore, pointing, acquisition, and tracking systems are essential for establishing and maintaining free-space optical communication links [7,8,9].

For miniaturized laser communication terminals, especially those designed for small satellites, unmanned platforms, or portable optical links, the pointing actuator must satisfy strict size, weight, and power constraints [10,11,12]. Traditional gimbal-based pointing mechanisms can provide a large angular range, but their volume, mass, mechanical complexity, and response speed are often unsuitable for highly integrated terminals. Fast steering mirrors can provide high pointing bandwidth, but the overall mechanical and optical assembly still limits further miniaturization. In this context, micro-electro-mechanical systems micromirrors are promising candidates for compact beam steering because they offer small size, low power consumption, fast response, and high integration potential [13,14].

MEMS micromirrors have been widely studied for optical scanning, display, LiDAR, biomedical imaging, and free-space optical links [15,16]. Depending on the actuation mechanism, MEMS mirrors can be driven electrostatically, electromagnetically, electrothermally, or piezoelectrically. Among these approaches, electrostatic actuation is attractive for space-constrained optical terminals because of its low static power consumption, simple structure, and compatibility with microfabrication processes. In laser communication systems, however, the MEMS micromirror is not only required to scan a large uncertainty region but also to perform stable quasi-static beam pointing after acquisition. This quasi-static operating mode places stricter requirements on the voltage–angle model, bidirectional control consistency, angular repeatability, and terminal-level calibration accuracy [17].

A key difficulty of electrostatic MEMS micromirror control is the nonlinear relationship between the driving voltage and the angular deflection [18,19]. The electrostatic torque is related to the square of the applied voltage and also depends on the angle-dependent capacitance gradient. Existing MEMS mirror studies have reported nonlinear modeling, quasi-static performance improvement, and model-based control. However, for a miniaturized laser communication terminal, the control problem is not limited to fitting the actuator response. The voltage command must be linked to the MEMS mechanical angle and then to the received optical spot position after terminal integration. Therefore, the device-level actuator nonlinearity and terminal-level optical calibration error should be treated separately.

Our previous work focused on MEMS-micromirror-based beam scanning and acquisition for miniaturized laser communication terminals, including the optical scanning model, scan trajectory design, overlap factor, and capture probability under platform microvibrations [20]. In contrast, the present work focuses on the actuator-level modeling and control problem. Instead of treating the MEMS micromirror as an ideal angular scanning device, this paper investigates how the measured nonlinear voltage–angle response can be modeled and compensated for accurate quasi-static beam pointing.

The specific contribution of this work is the integration of three practical steps for quasi-static MEMS beam pointing: differential-voltage actuation is used to obtain bidirectional control around a fixed bias voltage; a measured-data-based cubic static model is used to compensate the residual full-range voltage–angle nonlinearity that cannot be captured by a first-order linear model; and a two-level calibration framework is used to decouple the intrinsic MEMS voltage–angle response from the optical distortion, detector-coordinate rotation, and assembly errors introduced by the terminal.

The remainder of this paper is organized as follows. Section 2 introduces the MEMS micromirror and the miniaturized laser communication terminal configuration. Section 3 presents the nonlinear electrostatic-mechanical model and differential-voltage control principle. Section 4 describes the device-level and terminal-level calibration methods. Section 5 reports and discusses the experimental results, including voltage–angle fitting, dynamic response, QD-based calibration, and pointing verification. Section 6 concludes the paper.

## 2. Device and Terminal Configuration

### 2.1. Electrostatic MEMS Micromirror Under Test

The MEMS micromirror investigated in this work is a commercially available two-axis electrostatic quasistatic micromirror from Mirrorcle Technologies, Inc. (Richmond, CA, USA). The device identification number is S78879, and the manufacturer-defined actuator type is A8L2.2. It is a bonded aluminum mirror with a circular aperture of 5.0 mm and is packaged in a TINY48.4 package. The device operates in a gimbal-less dual-axis quasistatic mode, which is suitable for beam pointing applications where the mirror must be positioned at a commanded angle rather than driven continuously at resonance.

Unless otherwise stated, the angular values reported in this paper refer to the mechanical deflection angle of the MEMS mirror. The corresponding optical beam deflection angle is approximately twice the mechanical angle because of the reflection geometry.

The main measured parameters of the MEMS micromirror are summarized in Table 1.

The measured maximum mechanical angles indicate that the device can provide an optical steering range of approximately ±10° for each single axis before external optical magnification. In the proposed terminal architecture, the MEMS angular range is further expanded by the optical system to satisfy the field-of-view requirement of the miniaturized acquisition terminal.

### 2.2. Differential-Voltage Actuation Scheme

The MEMS micromirror is driven by electrostatic torque generated by opposite electrodes. For each axis, a bias voltage is applied to establish the operating point, and a differential voltage is used to generate bidirectional angular deflection. Taking the X axis as an example, the voltages applied to the two opposite electrodes can be expressed as

(1) $$V_{x+}=V_{b}+\frac{V_{dx}}{2}$$

(2) $$V_{x-}=V_{b}-\frac{V_{dx}}{2}$$

where $V_{b}$ is the bias voltage and $V_{dx}$ is the differential control voltage along the X axis. Similarly, the voltages for the Y axis can be written as

(3) $$V_{y+}=V_{b}+\frac{V_{dy}}{2}$$

(4) $$V_{y-}=V_{b}-\frac{V_{dy}}{2}$$

where $V_{dy}$ is the differential control voltage along the Y axis.

For an electrostatic actuator, the generated torque is proportional to the derivative of the capacitance with respect to the mirror angle and to the square of the applied voltage. Therefore, the net driving torque along one axis can be approximately expressed as the torque difference between two opposite electrodes:

(5) $$\tau_{x}\propto \frac{1}{2}\frac{\partial C_{x}}{\partial \theta_{x}}\left(V_{x+}^{2}-V_{x-}^{2}\right)$$

Substituting the differential-voltage expressions gives

(6) $$V_{x+}^{2}-V_{x-}^{2}={\left(V_{b}+\frac{V_{dx}}{2}\right)}^{2}-{\left(V_{b}-\frac{V_{dx}}{2}\right)}^{2}=2V_{b}V_{dx}$$

Thus, around a fixed bias voltage, the differential actuation scheme converts the voltage-squared electrostatic effect into a torque term that is approximately proportional to the differential command voltage. This is preferred over single-ended actuation for quasi-static pointing because it provides bipolar angular control around the bias point, avoids switching between opposite electrodes for positive and negative commands, improves command symmetry, and maintains higher small-signal sensitivity near the zero differential-voltage command. However, because the capacitance gradient, mechanical stiffness, and optical alignment are not perfectly linear over the full angular range, the measured relationship between $V_{d}$ and θ still exhibits nonlinear characteristics. Therefore, a nonlinear calibration model is required for accurate pointing control.

In this work, the measured static response is modeled by a polynomial relation between the differential voltage and the mechanical deflection angle:

(7) $$\theta_{i}=a_{i,3}V_{di}^{3}+a_{i,2}V_{di}^{2}+a_{i,1}V_{di}+a_{i,0},\;\;\;\;i\in \left\{x,y\right\}$$

where $V_{di}$ is the differential voltage command of the corresponding axis, and $\theta_{i}$ is the mechanical deflection angle in degrees. The coefficients $a_{i,3}$, $a_{i,2}$, $a_{i,1}$, and $a_{i,0}$ are obtained from the measured static response data.

The inverse relation from desired angle to differential voltage is then used to generate the control command for quasi-static pointing. The detailed model fitting and error analysis are presented in Section 3 and Section 5.

### 2.3. Miniaturized Laser Communication Terminal Architecture

**Figure 1:**
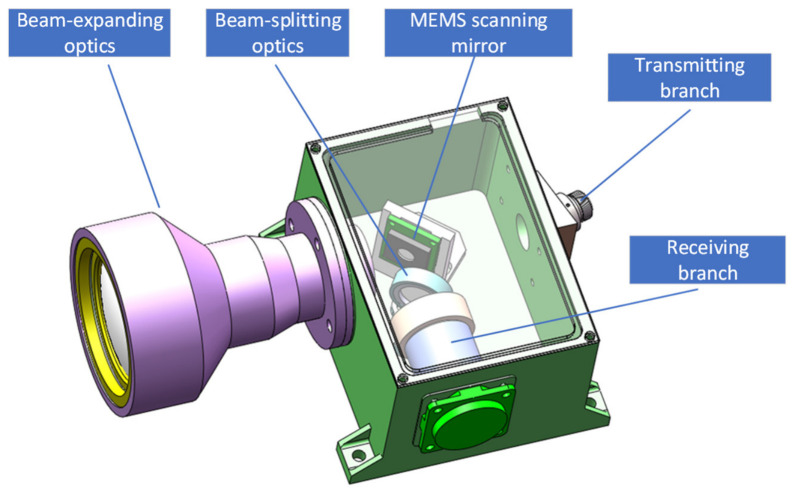
Design of miniaturized laser communication terminal.

The MEMS micromirror is integrated into a miniaturized laser communication terminal designed for compact beam scanning, acquisition, and pointing control. The terminal consists of an opto-mechanical module and an electronic control module. The opto-mechanical module includes the MEMS scanning mirror, beam-expanding optics, beam-splitting optics, a transmitting branch, and a receiving branch. The electronic module includes the laser driver, high-power amplifier, quadrant detector readout circuit, MEMS driver, and main control board. The terminal design is shown in Figure 1.

The optical design adopts a coaxial transmitting and receiving configuration. The received signal beam enters the terminal and is focused onto the active area of a quadrant detector through the receiving branch. The quadrant detector measures the spot position and provides feedback to the main controller. The controller then calculates the required correction command and drives the MEMS micromirror through the MEMS driver to adjust the outgoing or incoming beam direction.

The optical terminal uses a 3× beam-expanding component. For the receiving path, the beam-expanding optics compress the external acquisition field of view of 60° × 40° into an internal field of view of approximately 20° × 13.3°. For the transmitting path, the same optical magnification principle expands the MEMS steering range to support a large external scanning field. The terminal uses two optical wavelengths, 1540 nm and 1590 nm, for bidirectional operation. The transmitted beam divergence is approximately 0.25°.

A simplified control flow of the terminal can be described as follows: the incident beam is collected by the receiving optics and focused onto the quadrant detector; the four-channel detector voltages are converted into normalized spot coordinates; the spot-position error is calculated with respect to the detector center; the main controller converts the spot-position error into MEMS angular correction commands; the differential-voltage driver applies the corresponding voltages to the MEMS micromirror; and the mirror deflection changes the optical beam direction until the spot approaches the detector center.

This configuration enables the MEMS micromirror to function not only as a scanning actuator during acquisition, but also as a fine beam-pointing actuator during alignment and closed-loop tracking.

### 2.4. Quadrant-Detector Coordinate Definition

The quadrant detector generates four voltage outputs, denoted as $V_{A}$, $V_{B}$, $V_{C}$, and $V_{D}$. The normalized detector coordinates can be generally expressed as

(8) $$q_{x}=\frac{\left(V_{A}+V_{D})-(V_{B}+V_{C}\right)}{V_{A}+V_{B}+V_{C}+V_{D}}$$

(9) $$q_{y}=\frac{\left(V_{A}+V_{B})-(V_{C}+V_{D}\right)}{V_{A}+V_{B}+V_{C}+V_{D}}$$

Here, $q_{x}$ and $q_{y}$ represent the normalized spot displacement along the detector coordinate axes. The exact sign convention depends on the detector wiring, optical inversion, and terminal assembly direction. Therefore, the final mapping between the QD coordinate system and the MEMS angular coordinate system must be obtained through terminal-level calibration.

In the calibration experiment, one MEMS axis is driven while the other axis is kept nearly fixed. The corresponding four-channel QD voltages and spot coordinates are recorded. This procedure is repeated for both axes to determine the axis correspondence, coordinate sign, and nonlinear mapping between the MEMS mechanical angle and the QD spot position. The measured terminal data show that the MEMS deflection axes and the QD coordinate axes have a nearly orthogonal correspondence after alignment. However, large-angle operation still introduces noticeable distortion and installation-dependent errors, indicating that device-level voltage–angle calibration alone is insufficient for terminal-level beam pointing.

Therefore, this paper adopts a two-level calibration strategy. The first level is device-level calibration, which models the relationship between differential voltage and MEMS mechanical angle. The second level is terminal-level calibration, which maps MEMS angular deflection to QD spot displacement. By combining these two calibration levels, the complete control chain from voltage command to optical spot position can be established for the miniaturized laser communication terminal.

## 3. Nonlinear Electrostatic-Mechanical Modeling and Differential-Voltage Control

### 3.1. Electrostatic Actuation Principle

For an electrostatic MEMS micromirror, angular deflection is generated by the electrostatic torque between the movable mirror structure and the fixed driving electrodes. For one driving electrode, the electrostatic energy can be expressed as

(10) $$U_{e}=\frac{1}{2}C(\theta )V^{2}$$

where C(θ) is the angle-dependent capacitance between the movable and fixed electrodes, V is the applied voltage, and θ is the mechanical deflection angle of the micromirror. The corresponding electrostatic torque is obtained by differentiating the electrostatic energy with respect to the angular displacement:

(11) $$\tau_{e}=\frac{\partial U_{e}}{\partial \theta }=\frac{1}{2}\frac{\partial C(\theta )}{\partial \theta }V^{2}$$

This equation indicates that the driving torque is proportional to the square of the applied voltage and to the capacitance gradient. Therefore, even when the capacitance gradient is assumed to be constant near the neutral position, the voltage-to-torque relationship is inherently nonlinear for a single-ended electrostatic drive.

For bidirectional beam pointing, a pair of opposite electrodes is used for each axis. Taking the X axis as an example, the net electrostatic torque can be written as the torque difference generated by the two opposite electrodes:

(12) $$\tau_{x}=\frac{1}{2}\frac{\partial C_{x+}}{\partial \theta_{x}}V_{x+}^{2}-\frac{1}{2}\frac{\partial C_{x-}}{\partial \theta_{x}}V_{x-}^{2}$$

where $V_{x+}$ and $V_{x-}$ are the voltages applied to the positive and negative driving electrodes of the X axis, respectively. For an ideal symmetric actuator, the two capacitance gradients can be approximated as

(13) $$\frac{\partial C_{x+}}{\partial \theta_{x}}\approx \frac{\partial C_{x-}}{\partial \theta_{x}}=\Gamma_{x}(\theta_{x})$$

Thus, the net torque becomes

(14) $$\tau_{x}\approx \frac{1}{2}\Gamma_{x}(\theta_{x})(V_{x+}^{2}-V_{x-}^{2})$$

A similar expression can be obtained for the Y axis. This paired-electrode structure provides the physical basis for differential-voltage actuation.

### 3.2. Differential-Voltage Linearization

In the differential-voltage driving scheme, a fixed bias voltage is applied to both opposite electrodes, and the control command is introduced as a voltage difference. For the X-axis, the two driving voltages can be represented by Equations (1) and (2).

Substituting these two expressions into the voltage-square difference gives

(15) $$V_{x+}^{2}-V_{x-}^{2}=\left(V_{b}+\frac{V_{dx}}{2}\right)-\left(V_{b}-\frac{V_{dx}}{2}\right)=2V_{b}V_{dx}$$

Therefore, the net driving torque can be approximated as

(16) $$\tau_{x}\approx \Gamma_{x}(\theta_{x})V_{b}V_{dx}$$

Similarly, for the Y axis,

(17) $$\tau_{y}\approx \Gamma_{y}(\theta_{y})V_{b}V_{dy}$$

These equations show that, under a fixed bias voltage, the dominant differential driving torque is approximately proportional to the differential voltage command. This is the main advantage of differential-voltage control: it converts the voltage-squared actuation mechanism into a nearly linear bidirectional control form around the bias operating point.

However, this linearization is only an ideal approximation. In practice, $\Gamma_{i}(\theta_{i})$ is not constant over the full angular range. In addition, the mechanical restoring torque, mirror inertia, fabrication asymmetry, package stress, and weak axis coupling can all introduce nonlinear behavior. Therefore, the actual relationship between the differential voltage and the mirror angle must be identified experimentally.

### 3.3. Electrostatic-Mechanical Dynamic Model

For each axis of the MEMS micromirror, the angular motion can be described by a lumped rotational dynamic model:

(18) $$J_{i}{\ddot{\theta }}_{i}+c_{i}{\dot{\theta }}_{i}+k_{i}\theta_{i}+k_{3,i}\theta_{i}^{3}=\tau_{i}(V_{di},\theta_{i}),\;\;\;\;i\in \left\{x,y\right\}$$

where $J_{i}$ is the rotational inertia, $c_{i}$ is the damping coefficient, $k_{i}$ is the linear torsional stiffness, $k_{3,i}$ represents the nonlinear stiffness term, and $\tau_{i}$ is the electrostatic driving torque. The cubic stiffness term is included to describe the nonlinearity that becomes more evident at large angular deflection.

For small-signal motion around a fixed operating point, the dynamic response of each axis can be approximated by a second-order transfer function:

(19) $$G_{i}(s)=\frac{\Theta_{i}(s)}{V_{di}(s)}=\frac{K_{i}\omega_{n,i}^{2}}{s^{2}+2\varsigma_{i}\omega_{n,i}s+\omega_{n,i}^{2}}$$

where $K_{i}$ is the static angular sensitivity, $\omega_{n,i}=2\pi f_{n,i}$ is the natural angular frequency, and $\varsigma_{i}$ is the damping ratio. The damping ratio is related to the quality factor $Q_{i}$ by

(20) $$\varsigma_{i}=\frac{1}{2Q_{i}}$$

For the S78879 MEMS micromirror, the measured resonant frequencies are 317 Hz and 319 Hz for the X and Y axes, respectively. The measured quality factors are 23.9 and 25.5, corresponding to damping ratios of approximately 0.0209 and 0.0196. These parameters indicate that the device has a lightly damped mechanical response. The 120 Hz low-pass cutoff is approximately 0.38 times the measured resonant frequency, and is therefore used as a conservative command-shaping frequency to reduce excitation near the mechanical resonance. For quasi-static pointing, the effective command bandwidth is kept well below the resonant peaks; the 120 Hz filter should be understood as a resonance-suppression and command-smoothing measure rather than the final closed-loop bandwidth.

Using the measured resonance parameters, the normalized frequency ratio at 120 Hz is about 0.38. The corresponding second-order mechanical response is still below the resonant region, while command components close to 317–319 Hz would be strongly amplified because of the high Q factor. Therefore, the combination of bandwidth limitation and low-pass filtering is necessary for stable quasi-static pointing and moderate-speed acquisition control.

Because the present work focuses on quasi-static beam pointing for laser communication terminals, the static nonlinear voltage–angle relationship is more important than the high-frequency resonant behavior. The dynamic model is mainly used to determine the allowable control bandwidth and to avoid resonant excitation during fast pointing or scanning.

Although the differential-voltage driving scheme improves bidirectional controllability, the measured static response of the MEMS micromirror still shows evident nonlinear characteristics over the full angular range. The cubic polynomial used in this work is not intended to indicate that only cubic mechanical stiffness dominates the response. Instead, it is a compact static compensation model that captures the combined residual effects of angle-dependent capacitance gradient, electrostatic softening, nonlinear restoring torque, residual stress, fabrication asymmetry, and weak cross-axis coupling.

From a local expansion of the capacitance gradient and mechanical restoring torque around the nominal operating point, higher-order terms naturally appear in the voltage–angle relationship. A first-order model cannot describe the reduced angular sensitivity at large differential voltages, while a simple quadratic model is not appropriate for the biased differential-voltage form, whose dominant response is approximately odd-symmetric around the bias operating point. The cubic model is therefore selected as the lowest-order practical model that provides sufficient full-range fitting accuracy and remains convenient for inverse command generation.

For each axis, where $i\in \left\{x,y\right\}$ denotes the corresponding MEMS driving axis, the mechanical deflection angle is represented by the polynomial model in Equation (7).

### 3.4. Inverse Model for Differential-Voltage Command Generation

For beam pointing control, the input command is usually a desired angular deflection rather than a desired voltage. Therefore, the nonlinear voltage–angle model must be inverted to generate the required differential voltage.

Given a desired mechanical angle $\theta_{i,d}$, the corresponding voltage command $V_{di}$ can be obtained by solving

(21) $$a_{i,3}V_{di}^{3}+a_{i,2}V_{di}^{2}+a_{i,1}V_{di}+a_{i,0}-\theta_{i,d}=0$$

Because the operating range of the MEMS micromirror is limited and the measured voltage–angle curve is monotonic within the usable range, the physically meaningful solution can be selected from the real roots of the cubic equation. In practical implementation, the solution is further constrained by the maximum differential voltage:

(22) $$\left|V_{di}\right|\leq V_{d,\max}$$

For the S78879 device, the measured maximum differential voltages are approximately 179.01 V for the X axis and 177.55 V for the Y axis. Therefore, voltage saturation is applied in the controller to avoid over-driving the actuator.

Alternatively, an inverse polynomial can be directly fitted from the measured data:

(23) $$V_{di}=p_{i,3}\theta_{i}^{3}+p_{i,2}\theta_{i}^{2}+p_{i,1}\theta_{i}+p_{i,0}$$

The inverse-model coefficients identified from the measured data are

(24) $$V_{dx}=0.20763\theta_{x}^{3}-0.08482\theta_{x}^{2}+28.85217\theta_{x}+0.45163$$

(25) $$V_{dy}=0.20094\theta_{y}^{3}-0.09432\theta_{y}^{2}+29.39866\theta_{y}+0.12713$$

Here, the angle is in degrees and the voltage is in volts. In the subsequent experiments, the inverse model is used to generate the differential voltage command from the desired MEMS angular deflection. The direct cubic model is then used to evaluate the residual voltage–angle error.

### 3.5. Two-Level Control Mapping for Terminal Beam Pointing

In a miniaturized laser communication terminal, the MEMS voltage command is not the final control target. The final target is the optical spot position on the detector or the outgoing beam direction. Therefore, the complete pointing control chain contains two mapping levels.

The first level is the device-level mapping from differential voltage to MEMS mechanical angle:

(26) $$V_{di}\to \theta_{i}$$

This mapping is determined by the electrostatic-mechanical behavior of the MEMS micromirror and is modeled by the nonlinear voltage–angle calibration described above.

The second level is the terminal-level mapping from MEMS mechanical angle to detector spot position:

(27) $$\theta_{i}\to q_{j}$$

where $q_{j}$ represents the normalized coordinate measured by the quadrant detector. This mapping depends not only on the MEMS micromirror but also on the optical layout, beam-expanding optics, detector position, coordinate rotation, assembly error, and terminal alignment. Therefore, terminal-level calibration is required after the MEMS mirror is integrated into the optical terminal.

Combining the two mappings, the complete control chain can be written as

(28) $$V_{di}\to \theta_{i}\to q_{j}$$

For closed-loop beam pointing, the quadrant detector provides the spot-position error:

(29) $$e_{x}=q_{x,d}-q_{x}$$

(30) $$e_{y}=q_{y,d}-q_{y}$$

where $q_{x,d}$ and $q_{y,d}$ are the desired detector coordinates, usually corresponding to the detector center. The controller converts the detector-coordinate error into an angular correction command:

(31) $$\Delta \theta_{i}=F_{i}(e_{x},e_{y})$$

where $F_{i}(∙)$ is obtained from terminal-level calibration. The inverse voltage–angle model then converts the desired angular correction into a differential voltage command:

(32) $$V_{di}=M_{i}^{-1}(\theta_{i,d})$$

where $M_{i}^{-1}(∙)$ denotes the inverse nonlinear model of the MEMS micromirror.

This two-level mapping separates the intrinsic actuator nonlinearity from the terminal-level optical calibration error. As a result, the device-level model can be reused for the same MEMS micromirror, while the terminal-level calibration can be updated after optical assembly or realignment.

### 3.6. Control Strategy for Quasi-Static Beam Pointing

The proposed control strategy is designed for quasi-static beam pointing rather than resonant scanning. The main objective is to command the MEMS micromirror to a desired angular position with high repeatability and low residual error.

The control procedure is as follows.

First, the desired beam direction or detector spot position is converted into the desired MEMS mechanical angle through the terminal-level calibration model. Second, the desired mechanical angle is converted into a differential voltage command using the inverse nonlinear voltage–angle model. Third, the generated voltage command is limited by the maximum allowable differential voltage and filtered to avoid excitation of the mechanical resonance. Finally, the MEMS driver applies the corresponding differential voltages to the opposite electrodes of the micromirror.

For open-loop pointing, the command is directly generated from the calibrated inverse model. For closed-loop pointing, the quadrant-detector feedback is used to update the angular command iteratively until the spot-position error is reduced below a predefined threshold.

The overall control law can be expressed as

(33) $$\theta_{d,k+1}=\theta_{d,k}+K_{p}e_{k}+K_{i}\sum_{m=0}^{k}e_{m}$$

where $e_{k}$ is the detector-coordinate error at the k-th control step, and $K_{p}$ and $K_{i}$ are the proportional and integral gains. The resulting angular command is then converted into the differential voltage command using

(34) $$V_{d,k+1}=M^{-1}(\theta_{d,k+1})$$

Compared with a direct voltage-control approach, the proposed method has two advantages. First, the nonlinear inverse model compensates for the static voltage–angle nonlinearity of the MEMS micromirror. Second, the QD-based feedback compensates for terminal-level optical errors that cannot be eliminated by device-level calibration alone.

Therefore, the proposed modeling and control framework provides a practical solution for using electrostatic MEMS micromirrors as compact beam-pointing actuators in miniaturized laser communication terminals.

## 4. Calibration and Experimental Methods

### 4.1. Device-Level Static and Dynamic Characterization

The device-level characterization was performed to obtain the intrinsic voltage–angle response of the S78879 MEMS micromirror before terminal-level optical integration. The measured data include the static differential-voltage response, small-signal frequency response, and step response of both axes. The static response was used to identify the nonlinear voltage–angle model described in Section 3, while the dynamic response was used to determine the allowable command bandwidth.

For each axis, the differential voltage was swept within the usable operating range, and the corresponding mechanical deflection angle was recorded. A cubic polynomial model was fitted to the same measured by Equation (7).

The fitting accuracy was evaluated by the root-mean-square error and the maximum absolute error. The identified model was then inverted to generate the differential-voltage command from the desired mechanical angle.

Because the MEMS micromirror used in this work is a bonded mirror, the dynamic response was not treated as an ideal second-order system over the full frequency range. Bonded MEMS mirrors can exhibit more complex dynamic behavior because the mirror plate is assembled above the actuator plane, and approximate dynamic models are generally used only for bandwidth estimation and command filtering.

### 4.2. Terminal-Level QD Calibration

After optical integration, the MEMS mechanical angle is not directly equivalent to the spot position on the detector. Optical magnification, assembly error, detector offset, and coordinate rotation introduce an additional mapping between the MEMS angle and the quadrant-detector coordinate.

The prototype terminal adopts a coaxial transmitting and receiving optical configuration. The received beam is focused onto a quadrant detector, and the four detector voltages are used to calculate the spot position. The optical module includes a 3× beam-expanding component, which compresses a 60° × 40° external receiving field of view into an internal field of view of approximately 20° × 13.3°; in the transmitting path, the same optical component expands the MEMS scanning range to 60° × 40°. The terminal uses 1540 nm and 1590 nm laser beams, with an approximate divergence angle of 0.25°.

During calibration, one MEMS axis was driven while the other axis was kept fixed. The MEMS angle and QD coordinates were recorded simultaneously to determine the axis correspondence and nonlinear mapping. The corresponding mapping plots, including the fitted cubic relationships, are provided in Section 5 so that the fitting quality can be visually assessed. The measured terminal data show that the MEMS axes and QD coordinates are approximately orthogonal after alignment. For the calibrated prototype terminals, the QD-to-MEMS mapping was represented by cubic polynomials. For Terminal A,

(35) $$\theta_{A,x}=-0.8255q_{y}^{3}-0.008067q_{y}^{2}-0.7905q_{y}-0.003011$$

(36) $$\theta_{A,y}=1.04q_{x}^{3}+0.04856q_{x}^{2}+1.228q_{x}-0.02537$$

For Terminal B,

(37) $$\theta_{B,x}=-0.5968q_{y}^{3}-0.07801q_{y}^{2}-0.8918q_{y}-0.004605$$

(38) $$\theta_{B,y}=0.6323q_{x}^{3}+0.02313q_{x}^{2}+1.33q_{x}-0.001415$$

These calibration equations were obtained from the measured QD–MEMS correspondence and were used to convert detector-coordinate errors into MEMS angular correction commands.

### 4.3. Pointing Verification Method

The pointing verification experiment was designed to evaluate whether the calibrated MEMS micromirror could repeatedly steer the optical beam to a desired position. The verification included three steps.

First, the MEMS mirror was commanded to a series of angular positions, and the corresponding QD coordinates were recorded. This step verified the monotonicity and axis correspondence of the terminal-level calibration.

Second, large-angle steering was tested on a target plane to observe scan distortion introduced by the MEMS mirror and terminal optics. The measurement was used to distinguish terminal-level optical distortion from the intrinsic device-level voltage–angle nonlinearity.

Third, repeated pointing tests were conducted at a fixed working distance. The beam was repeatedly driven to the same target position, and the displacement of the spot on the target was recorded. The measured spot displacement was converted into an equivalent MEMS angular error according to the working distance.

This two-level verification method separates the device-level voltage–angle error from the terminal-level pointing error. The device-level model evaluates the intrinsic nonlinear response of the MEMS micromirror, while the terminal-level test evaluates the additional errors caused by optical integration, QD coordinate mapping, and assembly misalignment.

## 5. Results and Discussion

### 5.1. Static Voltage–Angle Fitting

**Figure 2:**
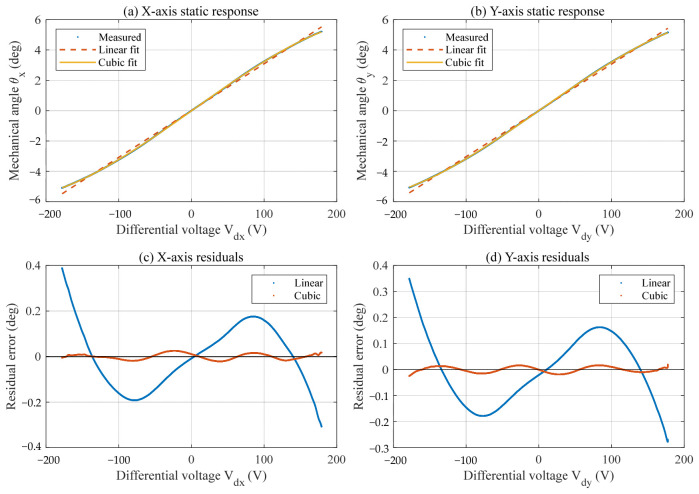
Measured static voltage–angle response and fitting residuals of the S78879 MEMS micromirror.

The measured static response of the S78879 MEMS micromirror was first used to evaluate the voltage–angle nonlinearity. Figure 2 shows the measured mechanical deflection angles and the fitted curves for both axes. Although the differential-voltage actuation scheme provides a nearly linear bidirectional driving form around the bias voltage, the full-range response still exhibits evident nonlinear deviation.

The fitting results are summarized in Table 2. For the X axis, the RMSE decreases from 0.142° with the linear model to 0.0127° with the cubic model. For the Y axis, the RMSE decreases from 0.132° to 0.0109°. The maximum fitting errors are also reduced from 0.388° to 0.0255° for the X axis and from 0.350° to 0.0246° for the Y axis. These results do not imply that polynomial fitting itself is a new method; rather, they show that a low-order cubic static compensation model is necessary and sufficient for the measured full-range differential-voltage response of this MEMS actuator.

### 5.2. Dynamic Response

In the present quasi-static pointing experiment, the filter is used for command shaping and resonance suppression rather than as a claim of closed-loop control bandwidth.

**Figure 3:**
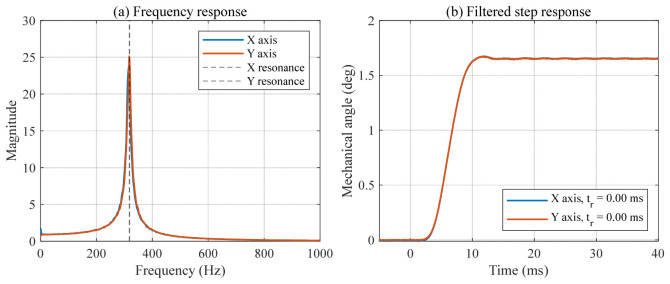
Step response of the X and Y axes under a differential-voltage command.

Figure 3 shows the step response under an approximately 50 V differential-voltage command. The measured 10–90% rise time is approximately 2.4 ms for both axes, and the angular response amplitude is about 1.65°. These results show that the MEMS micromirror has sufficient response speed for quasi-static beam pointing and moderate-speed acquisition control in a miniaturized laser communication terminal, provided that command components close to the resonant peaks are suppressed by bandwidth limitation and low-pass filtering.

### 5.3. Terminal-Level QD Calibration Results

**Figure 4:**
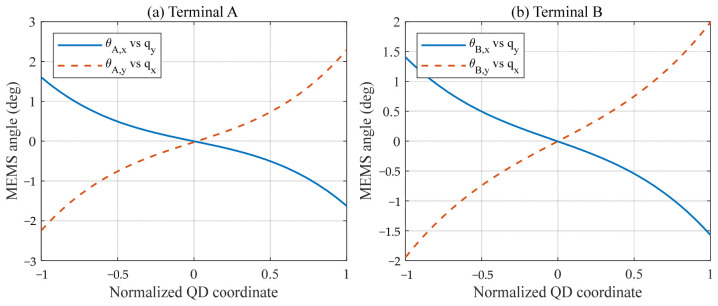
QD-coordinate-to-MEMS-angle calibration curves for Terminal A and Terminal B.

After the MEMS micromirror was integrated into the optical terminal, the QD-based calibration was used to establish the mapping between detector spot position and MEMS angular correction. Figure 4 shows the QD-coordinate-to-MEMS-angle calibration curves for the prototype terminals. The plots are included to show the mapping procedure and to allow visual assessment of the cubic calibration model used for terminal-level coordinate conversion.

The calibration results show that the MEMS angular axes and QD coordinate axes are approximately orthogonal after optical alignment. Specifically, MEMS X-axis deflection is mainly reflected in the $q_{y}$ coordinate, while MEMS Y-axis deflection is mainly reflected in the $q_{x}$ coordinate. This confirms the necessity of terminal-level coordinate calibration, because the detector-coordinate directions do not directly coincide with the MEMS electrical driving axes.

### 5.4. Large-Angle Distortion and Repositioning Accuracy

Large-angle steering was tested to evaluate the pointing distortion introduced by optical integration. At a working distance of 1 m, Terminal A shows an approximately 1 cm radial-direction deviation at full MEMS deflection, corresponding to an equivalent MEMS angular error of about 0.5°. The cross-axis deviation is about 0.5 cm, corresponding to about 0.28°. Terminal B shows larger distortion, with about 3 cm deviation at full deflection, corresponding to about 1.7° equivalent MEMS angular error. This larger error is likely related to installation and alignment deviations.

Repeated pointing tests were then conducted at a working distance of 3 m. Terminal A shows a repeated spot-position error of about 0.1 cm, corresponding to an equivalent angular repeatability of approximately 0.333 mrad, or 333 μrad/68.7 arcsec. Terminal B shows a repeated spot-position error of about 0.2 cm, corresponding to approximately 0.667 mrad, or 667 μrad/138 arcsec.

These results show that the intrinsic device-level voltage–angle fitting error is much smaller than the terminal-level pointing error. Therefore, the dominant error source after integration is no longer the MEMS static voltage–angle nonlinearity alone, but the combined effect of optical distortion, MEMS mounting error, detector-coordinate mapping, terminal assembly misalignment, and QD-related measurement uncertainty.

The above results verify the necessity of two-level calibration. The device-level cubic model compensates for the nonlinear relationship between differential voltage and MEMS mechanical angle, reducing the static fitting error to less than 0.03°. However, after the MEMS mirror is integrated into the optical terminal, large-angle distortion and coordinate misalignment introduce additional pointing errors. Therefore, QD-based terminal calibration is required to convert detector-coordinate errors into accurate MEMS angular correction commands.

For miniaturized laser communication terminals, this result has two implications. First, nonlinear voltage–angle modeling is necessary for accurate open-loop angular control of electrostatic MEMS micromirrors. Second, terminal-level optical calibration is indispensable for practical beam pointing, especially when the MEMS mirror is used over a large field of view. The proposed modeling and calibration framework separates these two error sources and provides a practical basis for subsequent closed-loop acquisition and tracking.

It should be noted that the present repeatability values include the combined influence of MEMS actuator repeatability, QD readout noise, optical stability, and mechanical alignment. Because QD noise was not independently isolated in this experiment, it is listed as an unseparated contribution in Table 3 rather than assigned an unsupported numerical value. Independent QD-noise characterization will be included in future closed-loop pointing experiments.

## 6. Conclusions

In this paper, nonlinear modeling and differential-voltage control of a two-axis electrostatic MEMS micromirror were investigated for miniaturized laser communication terminals. A 5.0 mm bonded aluminum MEMS micromirror was characterized at the device level, and its static voltage–angle response, resonant frequency, quality factor, and step response were analyzed. The differential-voltage actuation scheme was used to provide bidirectional quasi-static angular control around a fixed bias voltage, while the measured response showed that practical nonlinear compensation is still required over the full operating range.

A terminal-level calibration method based on a quadrant detector was further introduced to connect MEMS angular deflection with the optical spot position. The calibration results show that the MEMS driving axes and detector-coordinate axes are not directly coincident after optical integration, and that a separate QD-to-MEMS mapping is necessary. Large-angle steering and repeated pointing tests also indicate that terminal-level errors can be larger than the intrinsic voltage–angle fitting error of the MEMS micromirror. Therefore, accurate MEMS-based beam pointing requires both device-level nonlinear compensation and terminal-level optical calibration.

The contribution of this work is therefore not the use of a third-order polynomial alone, but the combination of differential-voltage actuation analysis, measured nonlinear static compensation, inverse voltage command generation, and two-level terminal calibration for a compact laser communication terminal. Future work will focus on real-time closed-loop pointing control, independent QD-noise characterization, and dynamic acquisition experiments under platform disturbances.

## Figures and Tables

**Table 1:** Main parameters of the MEMS micromirror under test.

Parameter	X Axis	Y Axis
Maximum differential voltage	179.01 V	177.55 V
Maximum mechanical angle	5.215°	5.161°
Resonant frequency	317 Hz	319 Hz
Quality factor	23.9	25.5

**Table 2:** Least-squares static voltage–angle fitting results obtained from the measured S78879 characterization data.

Axis	Model	Coefficients
X	Linear	$\theta_{x}=0.0308079V_{dx}+0.00830350$
Y	Linear	$\theta_{y}=0.03048147V_{dy}+0.02081994$
X	Cubic	$\theta_{x}=-1.60744\times 10^{-7}V_{dx}^{3}+1.86491\times 10^{-6}V_{dx}^{2}+0.03391498V_{dx}-0.01065501$
Y	Cubic	$\theta_{y}=-1.50112\times 10^{-7}V_{dy}^{3}+1.85584\times 10^{-6}V_{dy}^{2}+0.03336976V_{dy}+0.00051847$

**Table 3:** Summary of device-level and terminal-level error sources.

Error Source	Evaluation Method	Estimated Contribution
Device-level MEMS voltage–angle residual	Static cubic fitting residual	<0.03° for both axes
Large-angle terminal distortion	Target-plane deviation at 1 m	About 0.5° for Terminal A; about 1.7° for Terminal B
Repeated pointing error	Spot displacement at 3 m	Terminal A: ~333 μrad/68.7 arcsec; Terminal B: ~667 μrad/138 arcsec
QD noise and readout uncertainty	Included in repeatability measurement	Not independently isolated in the current experiment

## Data Availability

The data presented in this study are available on request from the corresponding author due to privacy.

## References

[1] Wang G., Yang F., Song J., Han Z. (2024). Free space optical communication for inter-satellite link: Architecture, potentials and trends. IEEE Commun. Mag..

[2] Cardakli M. (2025). Challenges and Opportunities in Free Space Optical Satellite Communication. J. Light. Technol..

[3] Raj A.B., Majumder A.K. (2019). Historical perspective of free space optical communications: From the early dates to today’s developments. IET Commun..

[4] Yao C.-K., Lin H.-P., Cheng C.-L., Li Y.-L., Du L.-Y., Peng P.-C. (2024). Satellite communication and free space optics for open radio access network. J. Light. Technol..

[5] Alhosani A., Alshehhi F., Almenhali M., Abu Hilal H. (2025). Optical communication advancements in free space and applications of free space orbital technology. J. Inst. Eng. Ser. B.

[6] Kaymak Y., Rojas-Cessa R., Feng J., Ansari N., Zhou M.C., Zhang T. (2018). A survey on acquisition, tracking, and pointing mechanisms for mobile free-space optical communications. IEEE Commun. Surv. Tutor..

[7] Chen L., Zhu L., Du H., Wang X., Shen S., Wang Y., Zhao S., Wang X. (2025). Pointing Acquisition and Tracking System for Free Space Optical Communication Based on Integrated Optical Phased Array. IEEE Photon-J..

[8] Nguyen T., Riesing K., Kingsbury R., Cahoy K. (2015). Development of a pointing, acquisition, and tracking system for a CubeSat optical communication module. Free-Space Laser Communication and Atmospheric Propagation XXVII.

[9] Chang J., Schieler C.M., Riesing K.M., Burnside J.W., Aquino K., Robinson B.S. (2019). Body pointing, acquisition and tracking for small satellite laser communication. Free-Space Laser Communications XXXI.

[10] Rödiger B., Roubal C., Rein F., Rüddenklau R., Vishwanath A.M., Schmidt C. (2025). OSIRIS4CubeSat—The World’s Smallest Commercially Available Laser Communication Terminal. Aerospace.

[11] Carrasco-Casado A., Shiratama K., Trinh P.V., Kolev D., Munemasa Y., Fuse T., Tsuji H., Toyoshima M. (2022). Development of a miniaturized laser-communication terminal for small satellites. Acta Astronaut..

[12] Sansone F., Francesconi A., Corvaja R., Vallone G., Antonello R., Branz F., Villoresi P. (2020). LaserCube optical communication terminal for nano and micro satellites. Acta Astronaut..

[13] Weihong Z., Yang W., Lihao W., Yichen L., Zhenyu W. (2023). Research progress of reliability of MEMS fast steering mirror for satellite laser communication. Infrared Laser Eng..

[14] Hou J., Yu H., Wu X., Shen W. Electromagnetically-Driven MEMS Fast Steering Mirror for Inter-Satellite Laser Communication. Proceedings of the 2025 IEEE 20th International Conference on Nano/Micro Engineered and Molecular Systems (NEMS).

[15] Fang X.-Y., Tu E.-Q., Zhou J.-F., Li A., Zhang W.-M. (2024). A 2D MEMS crosstalk-free electromagnetic micromirror for LiDAR application. J. Microelectromech. Syst..

[16] Ahmad M., Bahri M., Sawan M. (2024). MEMS micromirror actuation techniques: A comprehensive review of trends, innovations, and future prospects. Micromachines.

[17] Boulos D., Dupont E., Fracasso B., Al Hajjar H. (2025). Refined Dual-Step Calibration of MEMS Mirrors for Precision High-Resolution Beam Steering. IEEE Trans. Instrum. Meas..

[18] Zihao Y., Lihao W., Yang W., Yonggui Z., Yichen L., Zhenyu W. (2023). Control of a MEMS fast steering mirror with improved quasi-static performance. IEEE Access.

[19] Chai G., Tan Y., Tan Q., Dong R., Long X. (2023). Predictive gradient based control using Hammerstein model for MEMS micromirrors. IEEE/ASME Trans. Mechatron..

[20] Wang X., Han J., Wang C., Xie M., Liu P., Cao Y., Jing F., Wang F., Su Y., Meng X. (2023). Beam scanning and capture of micro laser communication terminal based on MEMS micromirrors. Micromachines.

